# Supplementary material for: A dynamic ensemble model for short-term forecasting in pandemic situations
Source: PLOS Glob Public Health. 2024 Aug 22;4(8):e0003058. doi: 10.1371/journal.pgph.0003058 (PMC11340948; doi:10.1371/journal.pgph.0003058)
Supplement: S1 Table — (PDF) [file pgph.0003058.s001.pdf]

Model	Hyperparameter	Search Space	Sampler
Random Forest	Min_samples_split<br>Min_samples_leaf	[2,4,8,16]<br>[1,2,4]	Grid-Search
XGBoost	Learning rate<br>Max_depth<br>Reg_lambda<br>Reg_alpha	(0.01,0.5)<br>(2,12)<br>(0,10)<br>(0,10)	TPE
LSTM	Learning rate<br>Batch size<br>Hidden state dim.<br># Hidden layers<br>Droprate	[0.001,0.005,0.01,0.05,0.1]<br>[16,32,64,128]<br>[16,32,64,128]<br>[1,2,3]<br>[0.0,0.2,0.5]	TPE
Meta-Model	Learning rate<br>Batch size<br># Hidden layers<br>Hidden state dim.	[0.001,0.005,0.01,0.05,0.1]<br>[16,32,64,128]<br>[0,1,2]<br>[8,16,32]	Grid Search
Meta-Model+LSTM*	Lookback Window<br>Learning rate<br>Batch size<br># Hidden layers<br>Hidden state dim.<br>Learning rate*<br>Hidden state dim.*<br># Hidden layers*<br>Droprate*	[2,3,4]<br>[0.001,0.005,0.01,0.05,0.1]<br>[16,32,64,128]<br>[0,1,2]<br>[8,16,32]<br>[0.001,0.005,0.01,0.05,0.1]<br>[4,8,16,32]<br>[1,2,3]<br>[0.0,0.2,0.5]	TPE

Table S1: Hyperparameters for base models and meta-models. The brackets correspond to discrete values, while the parentheses correspond to continuous parameter ranges. Hyperparameters denoted by a star belong to the LSTM model. The lookback window refers to the metadata. Hyperparameters were either optimized by using a grid-search or with a tree-structured Parzen estimator from optuna. As the base model's hyperparameters were tuned for each fitting window and dataset and the meta-model's hyperparameter for each dataset, we did not provide the final choices.
