# Supplementary figures and images for: A dynamic ensemble model for short-term forecasting in pandemic situations
Source: PLOS Glob Public Health. 2024 Aug 22;4(8):e0003058. doi: 10.1371/journal.pgph.0003058 (PMC11340948; doi:10.1371/journal.pgph.0003058)

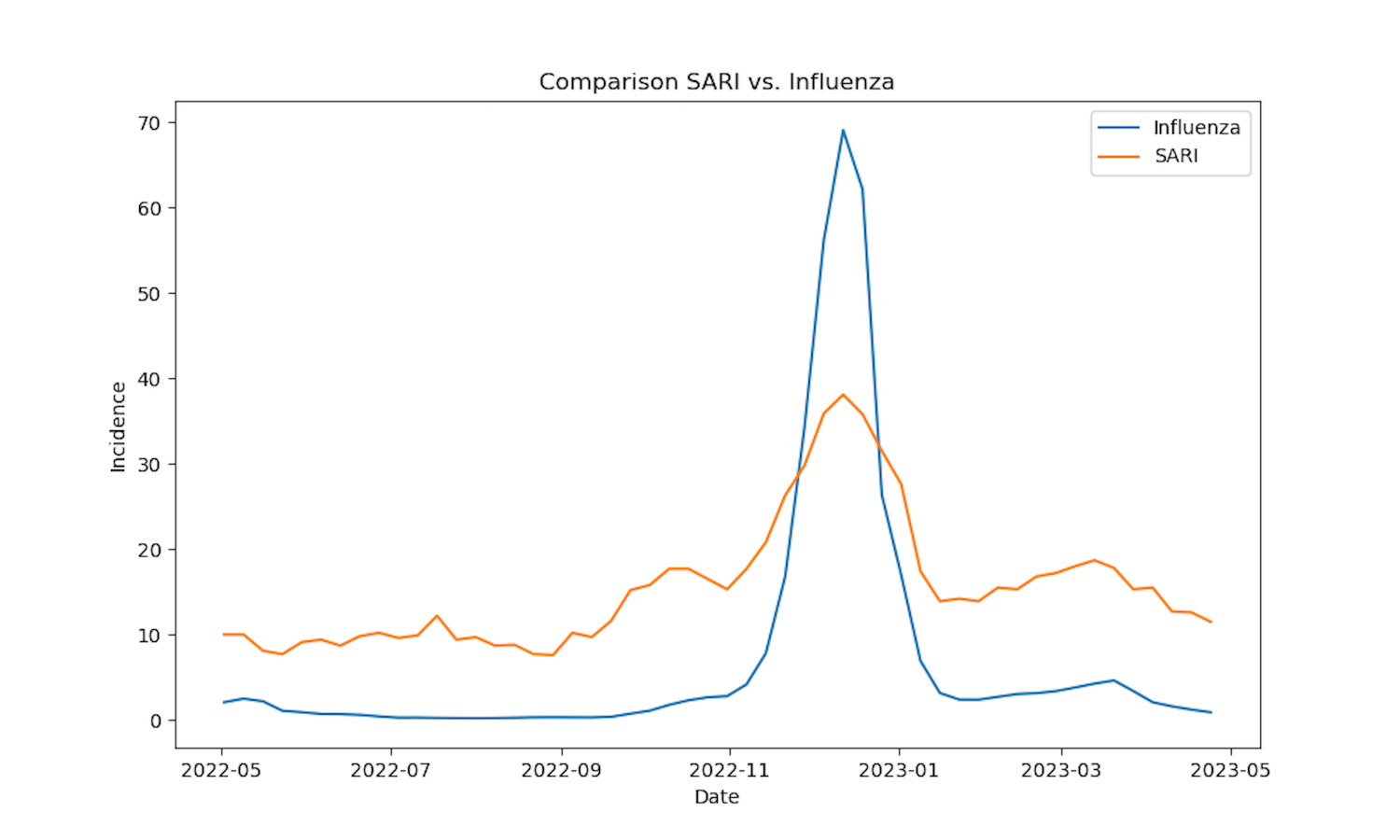

Supplement: S1 Fig — SARI and Influenza incidence from May 2022 to May 2023. (TIFF) [file pgph.0003058.s002.tiff]
