# Supplementary material for: A dynamic ensemble model for short-term forecasting in pandemic situations
Source: PLOS Glob Public Health. 2024 Aug 22;4(8):e0003058. doi: 10.1371/journal.pgph.0003058 (PMC11340948; doi:10.1371/journal.pgph.0003058)
Supplement: Attachment — Submitted filename: Response_to_Reviewers.docx [file pgph.0003058.s004.docx]

Dear Editor and Reviewers, we thank you for the valuable comments and critique. In the following we reply on the comments.

**Reviewer 1**

Major Comments:

**My strongest objection pertains to the concept of using autoregressive machine learning (AR ML) models to predict the epidemic of an emerging disease caused by an entirely new pathogen. While AR ML models may perform well in predicting smooth, predictable periods of an epidemic, as demonstrated by the authors, they necessarily fail at critical moments. Specifically, these models do not adequately address the system's response to planned restrictions, the height of epidemic waves: registered and more importantly the real ones, the dark figure (detection) coefficients, and the real derivatives of the former e.g. the real required number of hospital beds during peak demand periods.**

We agree that purely AR models (and also an ensemble of them) cannot account for exogenous factors influencing the dynamics of the pandemic directly as these information are not included as covariates. We modified our manuscript accordingly. We also added a discussion about the effect of the dark figure on our models performances. At the same time, we would like to point out that our paper focuses on short term-forecasting, where the influence of such exogenous factors is very limited, i.e. an almost stationary behavior can be expected. This is also reflected by the fact that explicit addition of meta-data (i.e. exogenous factors with potential influence) did not result into significantly improved prediction performances of our models.

**Selecting performance metrics based on the average performance across numerous segments, predominantly "smooth periods" with only occasional "tough periods," creates a misleading impression of the model's overall efficacy. For established diseases, this approach might be acceptable since high waves with high mortality are not typically expected. In such cases, precise predictions of stationary and regular time series, as seen in seasonal disease profiles (e.g., influenza and SARI in non-pandemic years), provide useful information to stakeholders.**

We agree that using the mean of the models performances can be misleading as outliers in critical moments are not represented well. We highlighted this in the manuscript, particularly in the limitation part of the conclusion. Additionally, we show in Section 3.5 on the example of the hospitalization that no significant increase in model prediction errors could be observed.

“We here only tested autoregressive base models, which may fail at critical turning points of the disease dynamics. Hence, in the future the inclusion of well calibrated mechanistic base models to our ensemble could be a recommendable strategy. Opposed to statistical and machine learning models, mechanistic models offer the possibility of counterfactual simulations for scenario planning. Furthermore, mechanistic models are often thought to be better understandable than machine learning models due to their explicit mathematical nature.”

**However, for non-stationary, emerging disease epidemics (such as the COVID-19 pandemic), AR ML models cannot be equated with other, more mechanistic and explainable models. Properly constructed mechanistic models (and some AI models based on a broader set of information) are developed on purpose to predict and explain non-stationary, reactive, complex, and socially significant periods in an epidemic. Contrary to the authors' statements in lines 49 and 50, these models are designed for such purposes.**

We do not want to generally argue for or against mechanistic models here, since we believe that AI/ML-based as well as mechanistic modeling strategies have their own merits and limitations. We also agree to the concern of non-stationary behavior, which is generally not well captured by statistical and ML models that are trained over long time periods. However, in our application scenario we train a machine learning model over short time intervals via a sliding window technique, and we focus on short-term forecasting, where a near stationary behavior can be expected, see our previous answer.

We adjusted the manuscript to highlight the strength and weaknesses of our approach, also in comparison to mechanistic models. In this context we also mentioned now that our proposed Dynamic Model Stacking approach could in the future also include well calibrated mechanistic models as part of the model ensemble.

**Using AR ML models to predict an emerging disease epidemic is akin to driving a car without fastening the seat belts. Most of the time, this approach may seem sufficient, but it fails at the accident. Mechanistic models, and other models that incorporate factors beyond autoregressive parameters, in this metaphor, function like seat belts: while they may be uncomfortable or costly to maintain during smooth periods, they have the potential to be effective during crises (e.g., the emergence of a new variant, reaching herd immunity, or the implementation of restrictions).**

We think that this comment re-iterates what has been mentioned before. We highlighted the principle limitations of AR approaches in our manuscript.

**### Detailed Explanation:**

**I realize that my main remark might be unsatisfactory for the authors; therefore, I provide a more detailed explanation of my point of view:**

**The underlying epidemic processes for these two groups of diseases (namely COVID-19 during the pandemic years and Influenza and SARI) are fundamentally different. COVID-19 was an emerging disease caused by a completely new pathogen, with no pre-existing immunity in any human population. This made the COVID-19 epidemic process entirely non-stationary from the onset until herd immunity was achieved. This is a switch like process, which was to some extend controlled by various mitigation policies. Additionally, the manifestation of this process in the observed number of cases, hospitalizations, and deaths was irregular, as these quantities were influenced not only by the epidemic process itself but also by random and unpredictable administrative changes in survey systems implemented independently by state and regional authorities.**

We completely agree . In our study we wanted to show that our model could be applied to COVID-19, influenza and SARI , despite that in their epidemic processes fundamentally differ.. We made the distinction and our aim clearer in the manuscript now.

**As I argued initially, using autoregressive ML models (broadly understood, including AR, MVAR, ML, and similar models) for predicting the course of an emerging disease is misleading unless it is explicitly stated that such models, by their construction, cannot forecast critical events in the pandemic's trajectory. Indeed, the accuracy of these models, averaged over all time segments, might be high and seemingly satisfactory. They perform well during relatively long periods of "smooth times" in a pandemic when no interventions are instituted, no new variants are present, and no changes in survey methods occur. However, during the most crucial moments, when decision-makers and resource managers need accurate predictions, autoregressive ML models inevitably fail. These models lack information about the system's potential response to new factors. In contrast, mechanistic models aim to predict system behaviour during non-stationary periods driven by new, previously unknown factors, such as the initiation of a vaccination campaign or the implementation of a strict lockdown.**

As already stated above we agree that purely AR models are not suitable to make forecast when dynamics change and impact the course of the pandemic. Also, they are not suitable for scenario planning as they do not account for confounding variables such as intervention effects, impact of emerging new variants. Still, we argue that AR models have been used in the literature (including COVID-19 short-term forecasting), because they are often independent of these changes when being applied to small segments of the time series, as here dynamics are nearly stationary. However, you are absolutely right, that this can unavoidably fail when non-stationary dynamics show their effect inside these segments or even at the boundary between training/fitting the data and evaluation. We better highlighted this limitation in our manuscript now.

**It is inaccurate, as the authors have stated, that mechanistic models are dependent on historical trend data (lines 49-50). Mechanistic models, in general, do not rely on historical trend data provided there is sufficient knowledge about the fundamental parameters they use. Importantly, mechanistic models do not require the assumption of process stationarity. The accuracy of well-constructed mechanistic models (such as Agent-Based Models) might be comparable or slightly weaker during "smooth times," but they are designed specifically to predict system reactions to factors mentioned by the authors (lines 50-52) without needing the assumption of stationarity.**

We elaborated on this:

“During that time many models were developed for short-term forecasting of the number of incident cases and hospitalizations, respectively [5], modeling strategies in this field include mechanistic, machine learning, and hybrid modeling strategies [5]. These models have been successful in capturing periods of the pandemic and for scenario planning. However, they often depend on fundamental parameters derived from historical data or expert knowledge. The highly dynamic nature of the pandemic, characterized by the rapid introduction of non-pharmaceutical interventions, new vaccines, and emerging virus variants, presented significant challenges for the generalizability of all forecasting models over extended periods. Consequently, models had to be frequently re-calibrated to stay aligned with the current situation.

**In summary, autoregressive models (including ML ensemble models) are suitable for predicting disease courses in their stationary phases (as demonstrated for SARI and influenza in non-pandemic years) or during "smooth times" within pandemic years. However, they are incapable of predicting crucial moments and factors (e.g., the height of a wave) for future pandemic developments during the emerging phase when biological or social interventions are applied. In theory, AI models might predict these moments but only by incorporating information from sources other than disease data time series, such as politicians' tendencies to institute a lockdown, which is extremely challenging to predict. While I do not discuss the quality and accuracy of different mechanistic models here, which can vary, I emphasize the essence of their construction and capabilities based on the information they incorporate. The degree of complexity of single AR ML model and the use of ensemble models do not change the situation.**

**The article focuses on short-term predictions, which might partially justify using AR ML models for emerging disease prediction. However, the fundamental limitations of such an approach must be discussed in more detail. In my opinion, the article is based on a misleading general idea that should be rephrased or thoroughly discussed.**

We agree to these points and want to emphasize again that our article focuses on short-term forecasting only. According to the criticism we have tried to make the assumptions and limitations of our modeling approaches clearer.

**Minor comments:**

1. ****Line 57:** "In principle, ensemble models can be understood as a collection of rather simplistic base models."**

***Comment:* Why describe the base models as simplistic? Each base model can be constructed very differently.**

. We removed the term “rather simplistic” from the manuscript.

2. ****Line 79:** "We incorporate six different datasets related to COVID-19."**

***Comment:* What does "incorporate" mean here? The crucial question is whether the model depends on a single variable or is multivariate. This is not clearly explained in the article. Specifically, does the prediction of incident cases rely solely on past incident cases, or does it include other time-series data (e.g., the number of deaths)? Please provide detailed description of input layer to the model.**

By incorporating we meant using these datasets as input for the models. All models were trained per indicator, i.e. we only used past confirmed incident cases to forecast confirmed incident cases, and only used past confirmed deaths to forecast confirmed deaths, etc. We changed the wording and added some additional information to explain this in more depth. Also, we included the training and testing dimensions and further explained their derivation in section 2.4.

3. ****Line 80:** "Additionally, we evaluated the models on weekly Influenza cases and weekly hospital admissions related to severe acute respiratory infections (SARI) in Germany."**

***Comment:* Similar to the previous remark, what data does each model depend on? Are these models single-variable autoregressive or multivariable autoregressive?**

See comment above.

4. ***Line 82:* "For this, we followed a sliding window approach (see Figure 1) with a training window size of 70 days."**

****Comment:** The training window is 70 days long, but what is the size of the input data?**

See comment above.

5. ****Line 84:** "The objective was to forecast the value of the time series 14 days ahead of time."**

***Comment:* The limitations of this objective need to be discussed. While this is suitable for short-term resource planning, it is not sufficient for long-term preparedness and intervention planning. More global measures are needed, such as wave height and the time of the wave peak.**

We agree that short-term forecasting is not suitable for long-term preparedness and intervention planning. We highlighted this limitation and the aim of our work in the manuscript.

6. ****Line 86:** "The MAPE represents the deviation of the prediction from the real data."**

***Comment:* These are not "real" data. It is important to distinguish between real cases and confirmed cases, as well as real (excess) deaths and confirmed deaths. This distinction is crucial in the epidemiological context. Predicting confirmed cases using ML and autoregressive models should be questioned, as the dark figure (the ratio between real and confirmed cases) depends on many factors, including administrative decisions, and cannot be accurately predicted using an autoregressive approach.**

Thank you for pointing us to this mistake. We changed the used term to confirmed and/or cases/deaths. Also, we added a discussion on the dark figure and how this impacts our models and the respective output.

**Reviewer 2**

1. **Impact of underreported cases: COVID-19 case reporting has significantly changed over time. During 2020-21, reporting was extensive, but it has relaxed as vaccines became popular and people increasingly neglected to report cases. Does this change, resulting in many underreported cases, affect your model's performance? In contrast, hospital admissions are likely less affected by underreporting. This could explain why models trained on hospital admissions (assuming these are for COVID-19; if not, please specify limitations) show lower MAPE. This point can be further validated by training models on data from the pandemic period (before 2022) to see if performance improves based on reported cases, assuming all cases were reported during that time. These concerns suggest that models trained on reported cases might not be suitable for the current situation. Have you tried forecasting the COVID situation in 2024 (if available) to check your method’s robustness and utility?**

We have added a discussion about the effect of the reporting on the performances of our models in the manuscript and also highlighted the differences between cases, deaths and hospitalisation in terms of data quality:

“The reporting of confirmed cases leads to the more general question of data quality. In Germany confirmed cases were reported by local health authorities to the RKI. However, measurement errors and underreporting might differ between those local health authorities and thus lead to discrepancies in data quality.

The definition of death due to COVID-19 can vary [48]. In the individual case it can be extremely challenging to identify a prior COVID-19 infection as the cause for death. Moreover, it is unlikely that all deaths during the pandemic were tested for a prior COVID-19 infection.

Similarly, hospitalization rates due to COVID-19 infection might suffer from a certain level of underreporting. However, we believe that the underreporting rate here was much lower than for confirmed cases. Therefore, we think that the better performance of the models in forecasting COVID-19 hospitalization could be due to higher data quality. “

Based on the suggestion, we also evaluated our best performing model during the time period August 2023 to June 2024. The results can be seen in section 3.4.

2. **The discussion on differences in model performance between daily and weekly datasets is insightful. Can you provide more information on why the Prev-Best model achieves better performance on daily data but fails on weekly data? Any additional results or explanations would be helpful.**

We have added some lines regarding this aspect in the manuscript:

“In general, we saw considerable differences between performances of models trained on data with a daily versus those trained on a weekly time resolution. Specifically, the SARI hospitalization data suggests that decision tree-based models - especially Random Forest - and the mean and median ensemble methods work well here. In this regard, we should point out that in the weekly data, the task is just to forecast the next two data points (2 weeks) which seems to be handled well by decision tree models. Linear regression struggles here because the model is fit to the past 5 data points (i.e. weeks), hence resulting in over-smoothing. Due to the low time resolution in the weekly datasets, base models may be overfitted and thus fail to predict subsequent test periods. In contrast, this effect is significantly reduced when training on data with a daily time resolution. We believe that this explains why the ensemble baseline model, Prev.-Best, which was among the top-performing models when trained on the daily data, whereas it showed significantly poorer performance when trained on the weekly data."

3. **The inclusion of metadata did not improve the model's performance. Is it necessary to include it in this paper?**

Even though, the inclusion of the metadata did not improve the model’s performance we still

believe that this finding is of interest and thus want to include it in the paper. We added some lines regarding this in the manuscript:

“The inclusion of Google Trends symptom counts as metadata could not further improve Dynamic Model Stacking significantly and thus cannot be recommended. We believe that metadata – such as Google Trends – could potentially improve model forecasts in other scenarios, since Google Trends data has been found to provide an early indicator of trends seen later on in surveillance data [24]. This aspect should be further investigated in future research.”

4. **In Table 1, does “regional (16+1)” refer to daily data from 16 regions plus weekly data at the country level? Please add table notes for clarity.**

We apologize for the confusion. The +1 stands for the country level data also on a daily
 resolution. We added table notes and modified the section (see section 2.1) to make it more clear.

5. **Line 177. Cite Table 1 and add descriptions to clearly explain what “N (140, 30, 80)” means.**

We added the citation and modified the section (see section 2.4) to explain what the N windows stand for.

6. **In Tables 3 and 5, highlight the p-values less than 0.01/0.001 instead of only showing the values.**

We modified the tables for better readability.
